# MRI T2 mapping assessment of T2 relaxation time in desmoid tumors as a quantitative imaging biomarker of tumor response: preliminary results

**DOI:** 10.3389/fonc.2023.1286807

**Published:** 2023-12-22

**Authors:** Felipe F. Souza, Gina D’Amato, Emily Elizabeth Jonczak, Philippos Costa, Jonathan C. Trent, Andrew E. Rosenberg, Raphael Yechieli, H. Thomas Temple, Pradip Pattany, Ty K. Subhawong

**Affiliations:** ^1^ Department of Radiology, Leonard M. Miller School of Medicine, University of Miami, Miami, FL, United States; ^2^ Sylvester Comprehensive Cancer Center, University of Miami Health System, Miami, FL, United States; ^3^ Department of Internal Medicine, Leonard M. Miller School of Medicine, University of Miami, Miami, FL, United States; ^4^ Department of Internal Medicine, Yale Medicine, New Haven, CT, United States; ^5^ Department of Pathology & Laboratory Medicine, Leonard M. Miller School of Medicine, University of Miami, Miami, FL, United States; ^6^ Department of Radiation Oncology, Leonard M. Miller School of Medicine, University of Miami, Miami, FL, United States; ^7^ Department of Orthopaedics, Leonard M. Miller School of Medicine, University of Miami, Miami, FL, United States

**Keywords:** desmoid-type fibromatosis, aggressive fibromatosis, magnetic resonance imaging, neoplasms, multiparametric magnetic resonance imaging, therapy response, soft tissue tumor

## Abstract

**Objectives:**

Because size-based imaging criteria poorly capture biologic response in desmoid-type fibromatosis (DF), changes in MRI T2 signal intensity are frequently used as a response surrogate, but remain qualitative. We hypothesized that absolute quantification of DF T2 relaxation time derived from parametric T2 maps would be a feasible and effective imaging biomarker of disease activity.

**Methods:**

This IRB-approved retrospective study included 11 patients with DF, managed by observation or systemic therapy, assessed by 3T MRI. Tumor maximum diameter, volume, and T2-weighted signal intensity were derived from manual tumor segmentations. Tumor:muscle T2 signal ratios were recorded. Two readers measured tumor T2 relaxation times using a commercial T2 scanning sequence, manual ROI delineation and commercial calculation software enabling estimation of reader reliability. Objective response rates based on RECIST1.1 and best responses were compared between size-based and signal-based parameters.

**Results:**

Median patient age was 52.6 years; 8 subjects were female (73%). Nine patients with longitudinal assessments were followed for an average of 314 days. Median baseline tumor diameter was 7.2 cm (range 4.4 - 18.2 cm). Median baseline T2 was 65.1 ms (range 40.4 - 94.8 ms, n=11); median at last follow-up was 44.3 ms (-32% from baseline; range 29.3 - 94.7 ms, n=9). T2 relaxation times correlated with tumor:muscle T2 signal ratios, Spearman p=0.78 (p<0.001). T2 mapping showed high inter-reader reliability, ICC=0.84. The best response as a percentage change in T2 values was statistically significant (mean -17.9%, p=0.05, paired t-test) while change in diameter was not (mean -8.9%, p=0.12).

**Conclusions:**

Analysis of T2 relaxation time maps of DF may offer a feasible quantitative biomarker for assessing the extent of response to treatment. This approach may have high inter-reader reliability.

## Highlights

- T2 relaxation time mapping at pixel resolution of desmoid fibromatosis enables achieving high inter-reader reliability (ICC=0.84)
- At best response, decreases in T2 values are larger as a percentage change from baseline than tumor diameter

## Introduction

Desmoid fibromatosis (DF) is an aggressive mesenchymal neoplasm that may cause substantial morbidity from infiltrative growth. Because of a propensity for local recurrence after resection, clinical management often involves observation or systemic therapy. Monitoring for disease progression and therapeutic response thus plays a critical role in influencing decisions to initiate or alter treatment regimens. Systemic therapy options including tyrosine kinase inhibitors, pazopanib, and gamma secretase inhibitors have all shown efficacy against DF in prospective studies (1–4). However, the primary imaging endpoints based on RECIST1.1 used in these studies are determined only by changes in maximum tumor diameter. Such criteria are ill-suited to capture biologic changes observed in DF, which is often marked by parenchymal collagenization, tumor shrinkage along minor axes, and a plateau in the decrease of tumor size so that attaining an objective response may take years. As a result, there is an emerging consensus that alternative DF-specific imaging response metrics are warranted that better reflect the clinical status of disease (5).

As DF undergoes collagenization, MRI signal intensity changes include increasing T2 hypointensity and decreased contrast-enhancement (6). Attempts to quantify the magnitude of such signal changes have traditionally utilized tumor to muscle signal ratios (7). T2 mapping is a commercially available quantitative MRI technique that permits absolute measurement of tissue T2 relaxation time, and has been shown to be sensitive to changes in collagen deposition (8). Quantitative assessment of T2 relaxation time in DF could improve objective tracking of treatment response by supplanting semantic descriptions of tumor signal changes and tumor:muscle signal ratios, which are heavily influenced by the region of reference muscle selected. Parametric measurements of tumor T2 relaxation time could allow more precise determination of tumor response and therefore be useful not only in routine clinical practice, but also clinical trial settings, where objective response rates and time to response are important efficacy endpoints. Because increasing collagenization is a hallmark of DF regression, we hypothesized that DF absolute T2 relaxation time derived from parametric T2 maps would be a feasible and effective imaging biomarker of disease activity. To determine this, we studied a retrospective cohort of DF patients to longitudinally quantify the change in T2 relaxation time and compare these measurements to changes in size and signal intensity ratios, and to compare the absolute T2 times between two readers.

## Methods

### Subjects

This retrospective study was approved by the local institutional review board with waiving of informed consent. MRI studies archived in the Picture Archiving Communication System (PACS, city) were reviewed, in addition to patient electronic medical records. Subjects were identified through keyword search of radiology reports since 2016 for “desmoid” OR “fibromatosis” OR “desmoid-type fibromatosis” AND “T2 map.” Inclusion criteria included subjects ≥18 years of age with histologically proven diagnosis of desmoid-type fibromatosis. Superficial fibromatosis cases were excluded. Subjects without T2 mapping sequences performed were excluded. The earliest MRI including the T2 mapping sequence was labeled “baseline MRI” regardless of preceding therapy or period of observation. The subject treatment history was recorded, including what regimens the patient was treated with for the study period.

### MRI protocol

**Figure 1:**
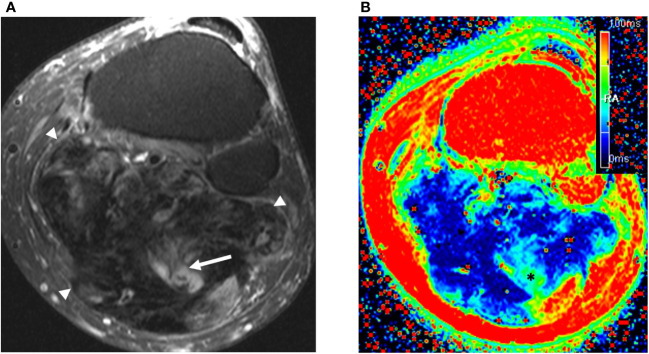
57 year old man with large calf mass. **(A)** Axial fat suppressed T2-weighted weighted MRI demonstrates a large mostly collagenized desmoid tumor in the calf (arrowheads). The tumor:muscle signal ratio was 0.34. Note small areas of internal T2 hyperintensity suggestive of more cellular components (arrow). **(B)** T2 mapping reveals low signal throughout the tumor, with areas of higher signal (*) corresponding with the T2 hyperintense components seen in **(A)** The T2 relaxation time of the tumor was measured by readers 1 and 2 as 33.6 ms and 29.3 ms, respectively.

All MRI examinations were carried out at 3T (Magnetom Verio and Skyra; Siemens, Erlangen, Germany). The local MRI protocol for desmoid tumors utilizes conventional T1 weighted turbo spin-echo, proton-density weighted turbo spin-echo with and without fat suppression, and a commercially available T2 mapping sequence (**Figure 1**). T2 mapping was performed using a multi-echo spin-echo technique, with variable echo times (TE’s) depending on anatomic region (**Table 1**). The T2 map was derived from a pixel-wise, mono-exponential non-negative least squares fit analysis (MapIt, Siemens Medical Solutions, Erlangen, Germany).

**Table 1:** T2 mapping parameters by anatomic region.

Region	TR/TEs (ms)	Slice (mm)	Matrix	NEX	Bandwidth (Hz/pixel)
Neck	3350/20, 40, 61, 81, 102	4	256 x 205	1	227
Thigh	3620/14, 28, 41, 55, 69	3	288 x 288	1	228
Abdominal Wall	2270-3010/14, 28, 41, 55, 69	3	256 x 205	1	225

MRI T2 mapping sequence parameters used for different anatomic regions. TR/TE, time to relaxation/time to echo; NEX, number of excitations; Hz, hertz.

### MRI assessment

Desmoid tumor maximum diameter (long axis), volume, and signal intensity were determined by semi-automated segmentation using dedicated software (mint Lesion™, v.3.6, mint medical, Dossenheim, Germany) by a fellowship trained musculoskeletal radiologist (TS, 11 years’ experience). Change in tumor size was categorized according to RECIST1.1 criteria of progressive disease (PD), partial response (PR), stable disease (SD), and complete response (CR) for a single target lesion (9).

To determine conventional tumor signal intensity, we adapted a modified Choi technique previously applied for measurements of soft tissue tumor MRI signal intensity (10). A two-dimensional region of interest (ROI) was constructed around the tumor using the manual segmentation tool in mint Lesion, using the fat-suppressed PD-weighted sequence at the slice showing the greatest tumor diameter. The signal intensity of nearby muscle tissue was also measured by placing an ROI with minimum 2 cm^2^ to serve as an internal reference, so that signal intensity could be normalized as a ratio of desmoid tumor to muscle signal on fluid sensitive sequences, similar to prior investigations (11, 12).

Single slice regions of interest (ROIs) on T2 maps were manually constructed using dedicated software (mint Lesion™, v.3.6, mint medical, Dossenheim, Germany); the slice depicting maximum cross-sectional tumor area was chosen for the ROI, analogous to the ROI placed on the fat-suppressed PD sequence above. A second fellowship trained musculoskeletal radiologist (FS, 12 years’ experience), blinded to the first reader’s measurements, independently constructed ROIs on the T2 maps to determine interobserver concordance.

### Statistics

Patient demographics were calculated descriptively, as were the clinical features of the individual tumors, including baseline tumor size and follow-up duration. Tumor response characteristics measured as changes in tumor diameter and tumor:muscle signal intensity ratios were compared to observed T2 relaxation times. The extent of response was calculated as the subject’s observed best response as a percentage change from baseline for both tumor diameter and T2 relaxation times, and were compared using a paired t-test after using a Shapiro–Wilk *W* test for normality. We used a natural logarithmic transformation of tumor:muscle signal intensity ratios since these ratios are distributed nonlinearly, with decreases bounded between 0 and 1, and increases in signal unbounded; we then correlated the ln (signal ratio) with quantitative T2 relaxation times. Inter-observer reliability between the two readers was calculated using intraclass correlation coefficient (ICC) for two-way random-effects model, absolute agreement; ICC values less than 0.5, between 0.5 and 0.75, greater than 0.75 up to 0.9, and greater than 0.90 were considered poor, moderate, good, and excellent reliability, respectively (13). Statistical analysis was performed using Stata 13.1 (StataCorp, College Station, TX, USA). For all analyses, results were considered statistically significant for p ≤ 0.05.

## Results

### Demographics and clinical characteristics

The study included 11 evaluable subjects, each with a single tumor; 8 subjects were female (73%). Mean age at baseline MRI was 52.9 years (median 52.6, range 27.0 – 78.7 years). Tumors were located in the chest wall (n=3), abdominal wall (n = 3), lower extremity (n =2), neck (n = 2), and upper extremity (n = 1). Tumor size at baseline varied considerably, with mean volume being 195 cm^3^, median 41 cm^3^, range 16 – 1,013 cm^3^. The mean tumor diameter (long axis) was 8.0 cm, median = 7.2 cm, range 4.4 to 18.2 cm.

### Pretreatment and therapy

Three tumors were locally recurrences after prior surgical excision. Most patients had been previously treated with one or two lines of therapy, either with surgical resection alone (n = 2), surgery plus systemic therapy (n = 1), or systemic therapy alone (n = 4); one subject had been heavily pre-treated with multiple modalities including systemic agents (tamoxifen, NSAIDS, tyrosine kinase inhibitors, irreversible electroporation, and liposomal doxorubicin). Three patients (27%) had not been previously treated.

Tumors were followed for an average of 314 days; two patients had only baseline scans available. A total of 37 timepoint assessments were performed. Therapies in use during the study interval included sorafenib 400 mg daily (n = 5), doxorubicin/dacarbazine (n = 1); two patients were enrolled in a placebo-controlled gamma-secretase clinical trial and investigators remain blinded to treatment arm. Three patients were undergoing active surveillance.

### Tumor response: RECIST

**Figure 2:**
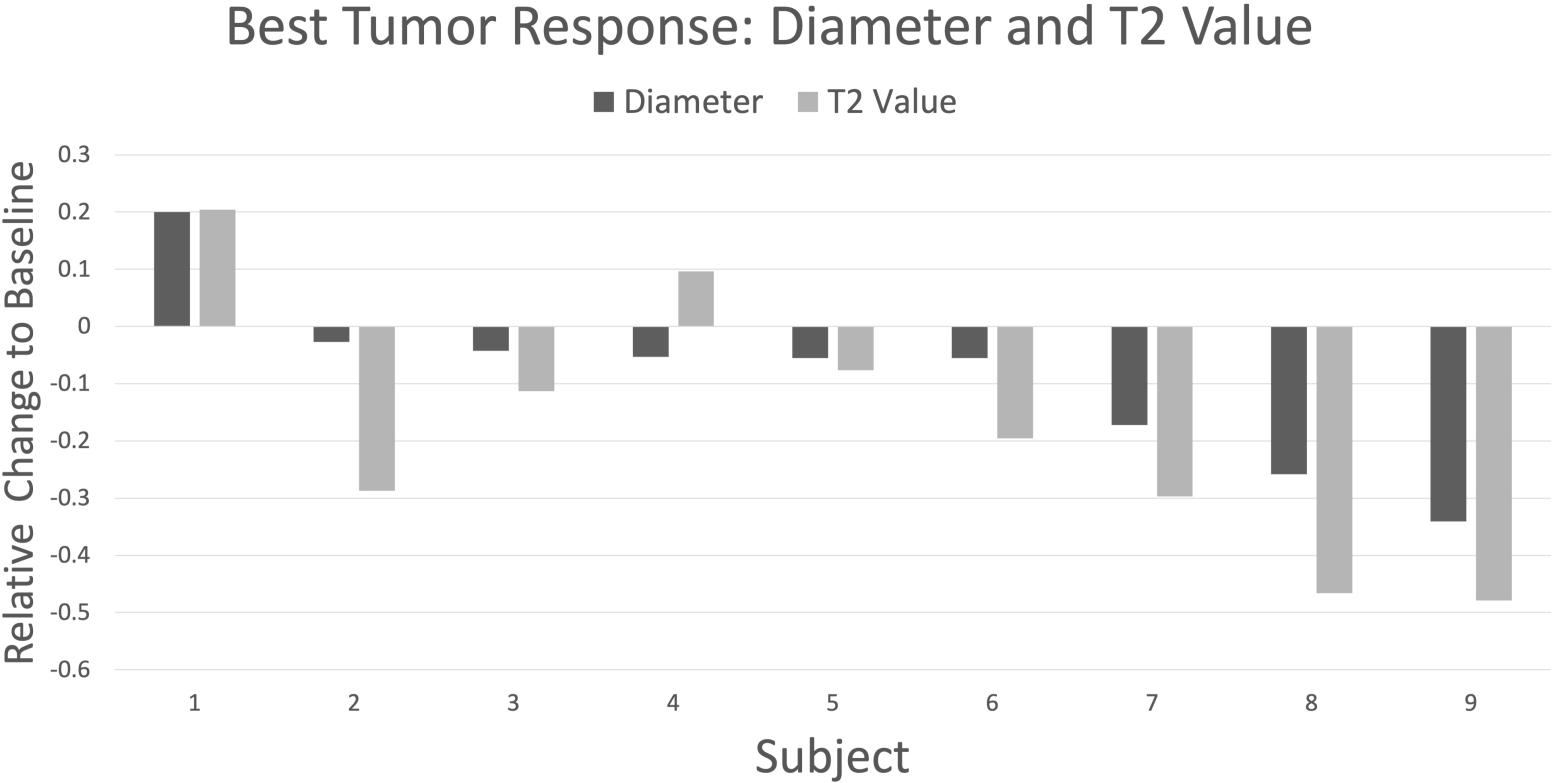
Waterfall plot depicting best response by change in maximum tumor diameter (dark gray) and T2 values (light gray) relative to baseline for nine subjects followed longitudinally. The plot is organized with higher subject numbers showing larger decreases in tumor diameter. There is good directional agreement with T2 values (r = 0.88, p = 0.002), with T2 values showing greater percentage change vs diameter in all cases except subject 4 (mean -17.9% vs -8.9%, respectively; p = 0.06, paired t-test).

**Figure 3:**
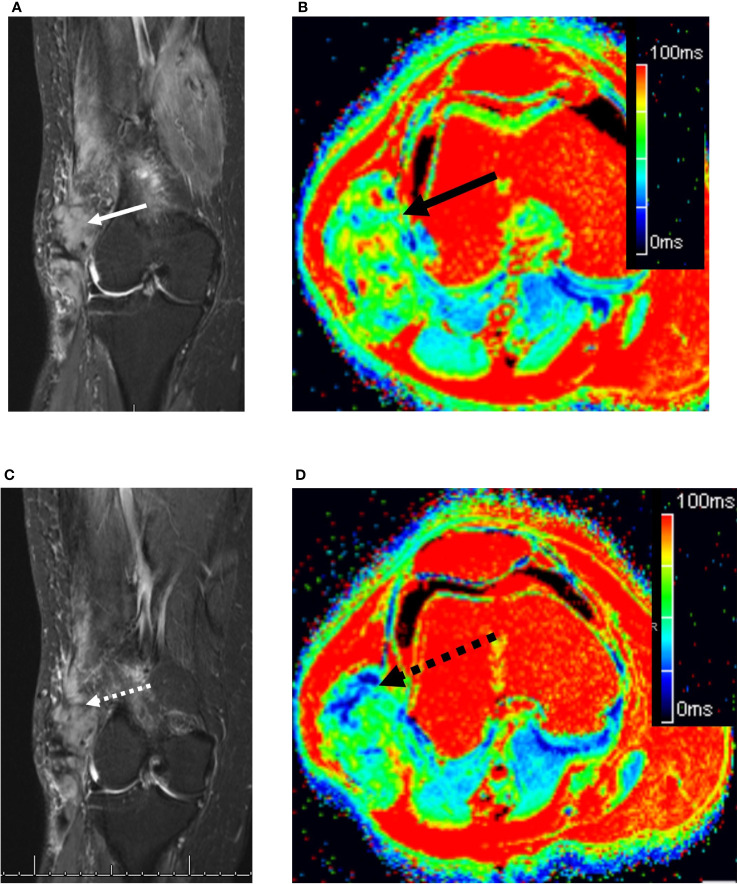
73 year old woman with a right lateral thigh mass. **(A)** Coronal fat-suppressed T2-weighted MRI demonstrates an ovoid heterogeneously hyperintense desmoid tumor in the lateral distal thigh (solid white arrow). **(B)** The corresponding axial T2 map demonstrated the mass (solid black arrow) to have a relaxation time of 59.1 ms (readers 1 and 2: 57.5 and 60.7 ms, respectively). **(C)** Four months later while on sorafenib, the tumor remains unchanged in length, but shows subtle decreased T2 hyperintensity (dashed white arrow). **(D)** Axial T2 map demonstrates small areas of decreased T2 relaxation times (dashed black arrow), bringing the mean tumor T2 down to 55.5 ms (readers 1 and 2: 56 and 54.9 ms, respectively).

Of 9 subjects with 26 follow-up timepoint assessments, tumor diameter decreased for 7 at the time of last follow-up, by an average of 13% (std dev 12%) (**Supplementary Figure 1**). The best response by tumor diameter is depicted in **Figure 2** as a waterfall plot. Only one subject achieved RECIST1.1 PR at a single timepoint, with a decrease in tumor diameter of 34% (from 4.4 to 2.9 cm). Six assessments among three patients were characterized as PD by RECIST1.1, with tumor diameter increases of up to 42%, 30%, and 28%, respectively. The remaining 19 timepoint assessments were characterized as RECIST1.1 SD, with a representative example shown in **Figure 3**.

### Tumor response: tumor:muscle T2 signal ratio and absolute T2 relaxation times

The mean tumor:muscle T2 signal ratio at baseline was 1.75 (median 1.55; range 0.49 to 4.62). There was a uniform decrease in signal ratios from baseline to last timepoint assessment, averaging -35.5% (median -41%; range -52% to -1.3%) (**Supplementary Figure 2**). Seven of the nine (78%) subjects showed at least a 30% reduction in T2 signal ratio; raw measurements of tumor and muscle T2 signal intensities is included as **Supplementary Table 1**.

The mean tumor T2 relaxation time was 61.4 ms at baseline, std dev = 17.7 ms (median 65.1 ms, range 40.4 - 94.8 ms, n=11). Of the nine subjects with serial examinations, the majority –7/9 (78%)—showed a decrease in T2 relaxation times, with mean value at last follow-up of 49.6 ms (std dev = 18.5 ms; median 44.3 ms, range 29.3 - 94.7 ms), for an average decrease of 9.5 ms (-15%; median -10.7 ms, range -26.3 to +9 ms) from baseline (**Supplementary Figure 3**). Tumor T2 relaxation times were highly correlated with tumor:muscle T2 signal ratios, Pearson r =0.71 (p < 0.001) (**Supplementary Figure 4**).

As can be seen the waterfall plot in **Figure 2** depicting best response by T2 relaxation time alongside best response by diameter, there was good directional agreement between T2 values and tumor diameter measurements (Pearson’s r = 0.88, p = 0.002). Because of the small sample size, a Shapiro-Wilk *W* test was performed and failed to show evidence of non-normality for best response differences in T2 (W = 0.95, p = 0.73) and diameter (W = 0.92, p = 0.32). Consequently, a parametric paired t-test was used to compare means. T2 values showed a statistically significant decrease in best response from baseline: mean -17.9%, p=0.05, paired t-test; however, the change in diameter was not statistically significant (mean -8.9%, p=0.12). Only for one subject out of nine was there discordance in best response, but this was relatively modest with the T2 value showing an increase of less than 10% from baseline; this subject exhibited RECIST1.1 SD throughout the study.

Two subjects showed increased T2 relaxation times at last follow-up: in one, from 45.6 to 54.9 ms (+20%), and corresponding RECIST PD with increase in diameter from 4.5 cm to 6.4 cm (+42%); in the other, from 40.4 to 44.3 ms (+9.6%), but had RECIST stable disease. T2 relaxation times at the six RECIST1.1 PD timepoints uniformly increased from baseline or corresponding nadir (mean 35% increase, range 13 to 85%), showing that parametric T2 maps are sensitive to the RECIST1.1 threshold of disease progression. The converse was also true, where the single RECIST1.1 PR timepoint in a different subject showed a corresponding 34% decrease in T2 (from 79.8 to 52.9 ms).

### T2 mapping inter-reader reliability

**Figure 4:**
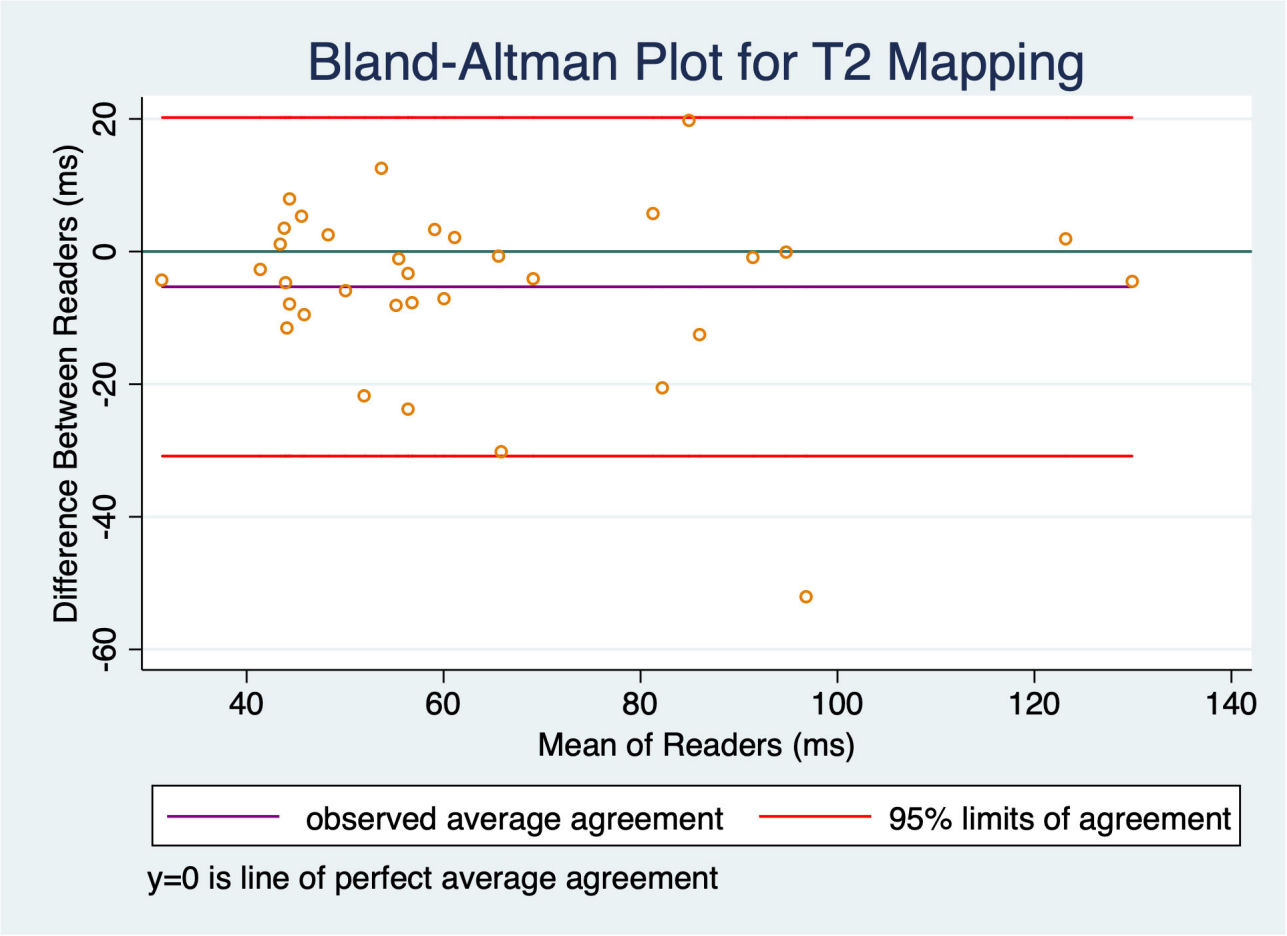
Bland-Altman plot shows slight bias of -5.3 ms, but this was not statistically significant as the confidence interval contains 0; there was good inter-reader reliability with an ICC of 0.84.

T2 mapping was deemed acceptable by both readers for 34 timepoints in the 11 subjects, with one reader recording technical failure of the sequence or tumors too small to reliably measure in 3/37 cases (8.1%). There was good correlation between readers for T2 mapping, with ICC = 0.84 (two-way random effects model, absolute agreement, individual measurements); this correlation was significant (F = 12.8, p < 0.001). As shown in the Bland-Altman plot (**Figure 4**), there was a small bias with an average difference between readers of 5.3 ms, std dev 13.0 ms, but this was not statistically significant (95% limits of agreement: -30.8 ms, 20.2 ms).

## Discussion

In this preliminary study, we show that commercially available T2 mapping (using a pixelwise, monoexponential nonnegative least-squares fit analysis) permits quantitative measurements of desmoid tumor T2 relaxation times, with high interobserver agreement, and compelling correlation with RECIST1.1 assessments. In particular, the extent of tumor response is higher when measured with T2 relaxation times as compared to tumor diameter. Extent (or depth) of response (best response as percentage change from baseline) has emerged as an important predictor of long term survival in other diseases (14), and our findings suggest that depth of T2 relaxation decrease may perform well as an imaging biomarker in the longitudinal assessment of desmoid tumors. As systemic therapy or active surveillance become established frontline management strategies for most DF, the Desmoid Tumor Working Group has identified the need for RECIST alternatives that are reliable and integrate “tissue response” into objective response criteria (5). Quantifying MRI signal changes by using T2 mapping shows promise in meeting this need, with high inter-reader reliability and correlation with changes in tumor size in this early study.

Previous prospective studies in phase II and III clinical trials have shown varying response rates of DF to systemic therapy. In a pool of 141 patients treated with imatinib, 1-year progression-free survival (PFS) was estimated to be 35-65%, 2-year PFS approximately 45%, and objective response rates 5-15% (15). Gounder et al., showed in a phase III trial that compared to placebo, sorafenib achieved significantly higher 2-year PFS (81% vs 36%), and higher objective response rates (33% vs 20%) (1). Toulmonde et al., showed objective PRs of 37% and 25% for pazopanib and methotrexate/vinblastine regimens, respectively (2). The most recent phase III trial of the gamma secretase inhibitor nirogacestat showed 41% of patients achieve RECIST objective responses, at a median time just under 6 months (16). Minimally invasive local therapies, either using cryoablation (17) or doxorubicin-eluting beads via transarterial chemoembolization (18), have also shown high success rates in achieving disease control.

However, it is clear that objective response rates systematically underestimate biologic response given high disease control rates and superior PFS compared to placebo. Establishing robust alternative response criteria could yield important practical advantages in clinical trial design and execution, among them an earlier determination of treatment efficacy based on objective imaging endpoints that would shorten trial duration. Using quantitative T2 values as a biomarker of biologic aggressiveness could lower screen failures due to overly stringent eligibility criteria—e.g. RECIST PD over a given interval, when *active* disease may be more appropriate. Cassidy et al. have shown that T2 hyperintensity involving >90% baseline tumor volume is associated with 1-year PFS of only 55%, compared to 94% in the <90% group (19); however, these were based on subjective estimates that could lead to lower inter-observer reliability. A quantitative T2 mapping strategy could enhance objective predictions of which tumors are most at risk for progression during active surveillance.

Any framework for desmoid tumor-specific alternative response criteria will likely take a multiparametric approach incorporating changes in both tumor size and signal (on T2-weighted or contrast-enhanced sequences) (20), although consensus threshold values have yet to emerge. Several prospective trials noted that alternative criteria performed well in secondary or exploratory analyses, and recent data has corroborated those initial insights: substantial decreases in tumor volume commonly precede RECIST PR by approximately 1 year on average (11). Moreover, volumetric analysis shows high inter-reader reliability, with ICC = 0.96, and the suggestion that the volumetric threshold for disease response or progression should be a change of 40% at minimum (21). We previously reported that patients who achieved RECIST PR or SD showed approximately 50% decreases in T2-signal ratio (7), a result echoed in a recent study where a 50% decrease was observed in 47% of patients (12). However, T2 signal ratios are problematic because the data is inherently asymmetric: increases are theoretically unbounded, while decreases are bounded between 0 and 1. Additionally, signal intensity in muscle tissue may be affected by technical artifact (e.g. inhomogeneous fat suppression, dielectric effects, etc.), or varying degrees of fatty infiltration and atrophy related to age and deconditioning, and thus an imperfect reference standard. DF radiomics has identified several features predictive of treatment response (11, 22), but may require post-acquisition correction for image signal heterogeneity, and arcane feature calculations often lack intuitive phenotypic meaning.

Morochnik et al., described changes in T2 mapping values in desmoid tumors treated with cryoablation. In those cases, increases in T2 relaxation time were observed, reflecting the cryoablation-induced liquefactive tumor necrosis (23). Similar increases in T2 hyperintensity have been observed in DF treated locally with cryoablation. In contrast, increases in tumor collagen deposition, either due to spontaneous disease regression or response to systemic therapy, are marked by *decreases* in tissue T2 relaxation time. Interestingly, a recent pilot study of intratumoral steroid injections showed significant decreases in DF T2 hyperintensity and contrast-enhancement quantified by tumor:muscle signal ratios (24). We have previously shown that T2 mapping is sensitive to collagenization in the superficial fibromatoses (25), and believe extending T2 mapping to DF is a rational imaging strategy that facilitates parametric analysis of the familiar DF-specific collagenization pattern that is typically described semantically, but challenging to quantify with traditional sequences.

This study has several limitations, the foremost being its retrospective nature with a small sample of convenience. Despite the small sample, we believe a sufficient number of timepoint assessments were available to demonstrate the feasibility of DF T2 mapping. Because patients were variously pre-treated and T2 mapping examinations were not uniformly obtained at true pre-therapeutic baseline, response thresholds cannot be proposed with confidence. However, this early data reveals strong sensitivity of the technique to changes in tumor signal over time, with high inter-reader agreement, which are both necessary characteristics of a robust response biomarker. Responses were not stratified by *CTNNB1* mutation status, but recent multi-institutional data suggests this does not influence response rates to systemic therapy (26). Additional limitations are the technical variability of the acquisitions, which revealed particular problems for DF in the abdominal wall due to respiratory motion artifacts. Though not represented in our data set, we suspect similar problems would create challenges for T2 mapping in mesenteric DF, at least as currently protocoled. Lastly, we utilized a two-dimensional ROI to measure mean T2 relaxation time on a single slice (where tumor diameter was maximum), rather than a volumetric segmentation on the T2 map. While this approach introduces sampling variability that could be particularly problematic for large heterogeneous tumors, it retains the chief advantage of simplicity, since it can be derived directly from the PACS, and is based on precedent where tumor: muscle signal ratios were similarly derived from a single representative slice (7, 27).

In conclusion, our preliminary results show that parametric T2 mapping sequences enable quantitative assessment of DF parenchymal characteristics with high inter-reader reliability. Utilizing this approach surmounts problems encountered with semantic descriptions and subjective estimates of tumor T2 hyperintensity, and eliminates the need for tumor signal ratios. Future studies could validate the approach with larger sample sizes, focus on establishing T2 mapping protocols for various anatomic regions, and assess whether volumetric analysis or T2 map radiomics could yield additional biomarkers of tumor behavior and therapeutic response.

## Data availability statement

The raw data supporting the conclusions of this article will be made available by the authors, without undue reservation.

## Ethics statement

The studies involving humans were approved by University of Miami Institutional Review Board. The studies were conducted in accordance with the local legislation and institutional requirements. The ethics committee/institutional review board waived the requirement of written informed consent for participation from the participants or the participants’ legal guardians/next of kin because the research involves no more than minimal risk to subjects.

## Author contributions

FS: Conceptualization, Investigation, Writing – original draft, Writing – review & editing. GD: Conceptualization, Investigation, Writing – review & editing, Funding acquisition. EJ: Conceptualization, Investigation, Writing – review & editing. PC: Writing – review & editing, Data curation. JT: Writing – review & editing, Conceptualization, Resources. AR: Conceptualization, Writing – review & editing, Data curation. RY: Conceptualization, Writing – review & editing. TT: Conceptualization, Writing – review & editing. PP: Conceptualization, Writing – review & editing, Investigation, Methodology, Resources, Software. TS: Conceptualization, Investigation, Methodology, Writing – review & editing, Data curation, Formal analysis, Project administration, Supervision, Writing – original draft.

## Supplementary material

Supplementary Figure 1 Subject level (n=9) changes in maximum tumor diameter from baseline to last exam, showing modest decrease for most subjects (mean -13%).

Supplementary Figure 2 Subject level (n=9) changes in tumor:muscle signal ratio from baseline to last exam, showing decrease for most subjects (mean -36%).

Supplementary Figure 3 Subject level (n=9) changes in T2 relaxation time from baseline to last exam, showing decrease for most subjects (mean -15%).

Supplementary Figure 4 Scatter plot depicting T2 Relaxation time vs tumor:muscle signal ratio across 36 time- point assessments shows good correlation (Pearson r =0.71, p < 0.001).

## References

[1] Gounder MM Mahoney MR Van Tine BA Ravi V Attia S Deshpande HA. Sorafenib for advanced and refractory desmoid tumors. N Engl J Med (2018)379(25):2417–28. 10.1056/NEJMoa1805052 PMC6447029 30575484

[2] Toulmonde M Pulido M Ray-Coquard I Andre T Isambert N Chevreau C. Pazopanib or methotrexate-vinblastine combination chemotherapy in adult patients with progressive desmoid tumours (DESMOPAZ): a non-comparative, randomised, open-label, multicentre, phase 2 study. Lancet Oncol (2019) 20(9):1263–72. 10.1016/S1470-2045(19)30276-1 31331699

[3] Kummar S O’Sullivan Coyne G Do KT Turkbey B Meltzer PS Polley E. Clinical activity of the γ-secretase inhibitor PF-03084014 in adults with desmoid tumors (Aggressive fibromatosis). J Clin Oncol Off J Am Soc Clin Oncol (2017) 35(14):1561–9. 10.1200/JCO.2016.71.1994 PMC5455706 28350521

[4] Greene AC Van Tine BA. Are the pieces starting to come together for management of desmoid tumors? Clin Cancer Res Off J Am Assoc Cancer Res (2022) 22:0620. 10.1158/1078-0432.CCR-22-0620 35819317

[5] Desmoid Tumor Working Group. The management of desmoid tumours: A joint global consensus-based guideline approach for adult and paediatric patients. Eur J Cancer Oxf Engl (1990) 127:96–107. 10.1016/j.ejca.2019.11.013 32004793

[6] Ganeshan D Amini B Nikolaidis P Assing M Vikram R. Current update on desmoid fibromatosis. J Comput Assist Tomogr. (2019) 43(1):29–38. 30211798 10.1097/RCT.0000000000000790 PMC6331223

[7] Sheth PJ Del Moral S Wilky BA Trent JC Cohen J Rosenberg AE. Desmoid fibromatosis: MRI features of response to systemic therapy. Skeletal Radiol (2016) 45(10):1365–73. 10.1007/s00256-016-2439-y 27502790

[8] Fukawa T Yamaguchi S Watanabe A Sasho T Akagi R Muramatsu Y. Quantitative assessment of tendon healing by using MR T2 mapping in a rabbit achilles tendon transection model treated with platelet-rich plasma. Radiology (2015) 276(3):748–55. 10.1148/radiol.2015141544 25816105

[9] Eisenhauer EA Therasse P Bogaerts J Schwartz LH Sargent D Ford R. New response evaluation criteria in solid tumours: revised RECIST guideline (version 1.1). Eur J Cancer Oxf Engl (2009) 45(2):228–47. 10.1016/j.ejca.2008.10.026 19097774

[10] Stacchiotti S Collini P Messina A Morosi C Barisella M Bertulli R. High-grade soft-tissue sarcomas: tumor response assessment–pilot study to assess the correlation between radiologic and pathologic response by using RECIST and Choi criteria. Radiology (2009) 251(2):447–56. 10.1148/radiol.2512081403 19261927

[11] Subhawong TK Feister K Sweet K Alperin N Kwon D Rosenberg A. MRI volumetrics and image texture analysis in assessing systemic treatment response in extra-abdominal desmoid fibromatosis. Radiol Imaging Cancer. (2021) 3(4):e210016. 34213370 10.1148/rycan.2021210016 PMC8344342

[12] Zanchetta E Ciniselli CM Bardelli A Colombo C Stacchiotti S Baldi GG. Magnetic resonance imaging patterns of tumor response to chemotherapy in desmoid-type fibromatosis. Cancer Med (2021) 10(13):4356–65. 10.1002/cam4.3973 PMC8267164 34102009

[13] Koo TK Li MY. A guideline of selecting and reporting intraclass correlation coefficients for reliability research. J Chiropr Med (2016) 15(2):155–63. 10.1016/j.jcm.2016.02.012 PMC4913118 27330520

[14] Cremolini C Loupakis F Antoniotti C Lonardi S Masi G Salvatore L. Early tumor shrinkage and depth of response predict long-term outcome in metastatic colorectal cancer patients treated with first-line chemotherapy plus bevacizumab: results from phase III TRIBE trial by the Gruppo Oncologico del Nord Ovest. Ann Oncol Off J Eur Soc Med Oncol (2015) 26(6):1188–94. 10.1093/annonc/mdv112 25712456

[15] Kasper B Gruenwald V Reichardt P Bauer S Rauch G Limprecht R. Imatinib induces sustained progression arrest in RECIST progressive desmoid tumours: Final results of a phase II study of the German Interdisciplinary Sarcoma Group (GISG). Eur J Cancer Oxf Engl (2017) 76:60–7. 10.1016/j.ejca.2017.02.001 28282612

[16] Gounder M Ratan R Alcindor T Schöffski P van der Graaf WT Wilky BA. Nirogacestat, a γ-secretase inhibitor for desmoid tumors. N Engl J Med (2023) 388(10):898–912. 36884323 10.1056/NEJMoa2210140 PMC11225596

[17] Mandel JE Kim D Yarmohammadi H Ziv E Keohan ML D’Angelo SP. Percutaneous cryoablation provides disease control for extra-abdominal desmoid-type fibromatosis comparable with surgical resection. Ann Surg Oncol (2022) 29(1):640–8. 10.1245/s10434-021-10463-7 PMC9391920 34269943

[18] Kim D Keohan ML Gounder MM Crago AM Erinjeri JP. Transarterial chemoembolization with doxorubicin eluting beads for extra-abdominal desmoid tumors: initial experience. Cardiovasc Intervent Radiol (2022) 45(8):1141–51. 10.1007/s00270-022-03149-4 PMC9400546 35441242

[19] Cassidy MR Lefkowitz RA Long N Qin LX Kirane A Sbaity E. Association of MRI T2 signal intensity with desmoid tumor progression during active observation: A retrospective cohort study. Ann Surg (2018) 271(4):748–755. doi: 10.1097/SLA.0000000000003073 PMC6736761 30418203

[20] Braschi-Amirfarzan M Keraliya AR Krajewski KM Tirumani SH Shinagare AB Hornick JL. Role of imaging in management of desmoid-type fibromatosis: A primer for radiologists. Radiogr Rev Publ Radiol Soc N Am Inc. (2016) 36(3):767–82. 10.1148/rg.2016150153 27163593

[21] Gondim Teixeira PA Biouichi H Abou Arab W Rios M Sirveaux F Hossu G. Evidence-based MR imaging follow-up strategy for desmoid-type fibromatosis. Eur Radiol (2020) 30(2):895–902. 31468156 10.1007/s00330-019-06404-4

[22] Crombé A Kind M Ray-Coquard I Isambert N Chevreau C André T. Progressive desmoid tumor: radiomics compared with conventional response criteria for predicting progression during systemic therapy-A multicenter study by the french sarcoma group. AJR Am J Roentgenol. (2020) 215(6):1539–48. 10.2214/AJR.19.22635 32991215

[23] Morochnik S Ozhinsky E Rieke V Bucknor M. T2-mapping as a predictor of non-perfused volume in MRgFUS treatment of desmoid tumors. Int J Hyperth Off J Eur Soc Hyperthermic Oncol North Am Hyperth Group (2019) 36(1):1272–7. 10.1080/02656736.2019.1698773 PMC6992380 31822140

[24] Wilke BK Garner HW Bestic JM Chase LA Heckman MG Schoch JJ. A pilot study of intralesional injection of triamcinolone acetonide for desmoid tumors: two-year outcomes. Clin Cancer Res Off J Am Assoc Cancer Res (2023) 29(3):541–7. 10.1158/1078-0432.CCR-22-2732 36455003

[25] Ramachandran A Fox T Wolfson A Banks J Subhawong TK. Superficial fibromatosis: MRI radiomics and T2 mapping correlate with treatment response. Magn Reson Imaging. (2021) 81:53–9. 10.1016/j.mri.2021.06.003 34116132

[26] Nathenson MJ Hu J Ratan R Somaiah N Hsu R DeMaria PJ. Systemic chemotherapies retain anti-tumor activity in desmoid tumors independent of specific mutations in CTNNB1 or APC: A multi-institutional retrospective study. Clin Cancer Res Off J Am Assoc Cancer Res (2022) 28(18):4092–4104. 10.1158/1078-0432.CCR-21-4504 PMC9475245 35180772

[27] Gounder MM Lefkowitz RA Keohan ML D’Adamo DR Hameed M Antonescu CR. Activity of Sorafenib against desmoid tumor/deep fibromatosis. Clin Cancer Res Off J Am Assoc Cancer Res (2011) 17(12):4082–90. 10.1158/1078-0432.CCR-10-3322 PMC3152981 21447727

