# Supplementary figures and images for: MRI T2 mapping assessment of T2 relaxation time in desmoid tumors as a quantitative imaging biomarker of tumor response: preliminary results
Source: Front Oncol. 2023 Dec 22;13:1286807. doi: 10.3389/fonc.2023.1286807 (PMC10766853; doi:10.3389/fonc.2023.1286807)

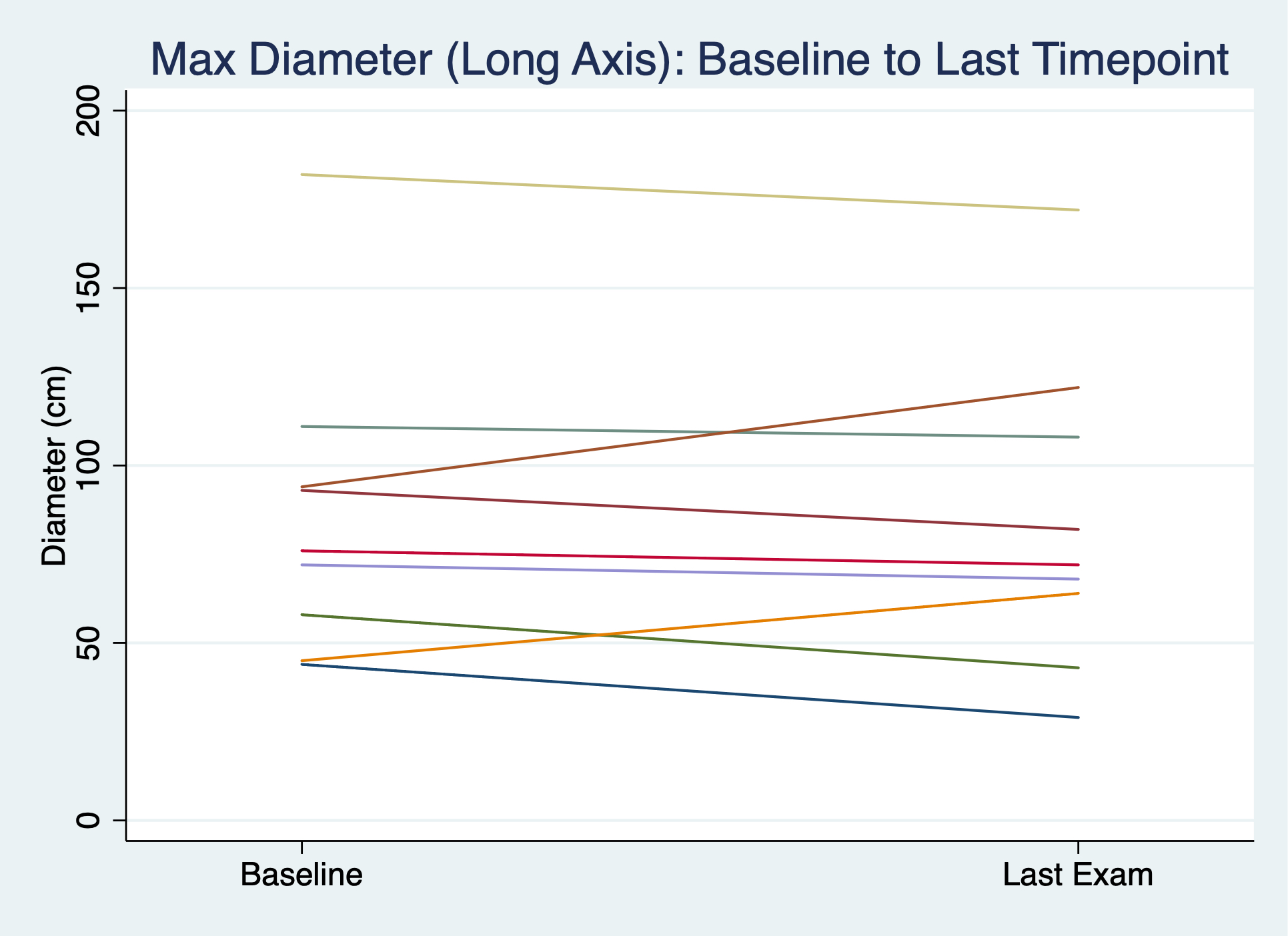

Supplement: Supplementary Figure 1 — Subject level (n=9) changes in maximum tumor diameter from baseline to last exam, showing modest decrease for most subjects (mean -13%). [file Image_1.jpeg]

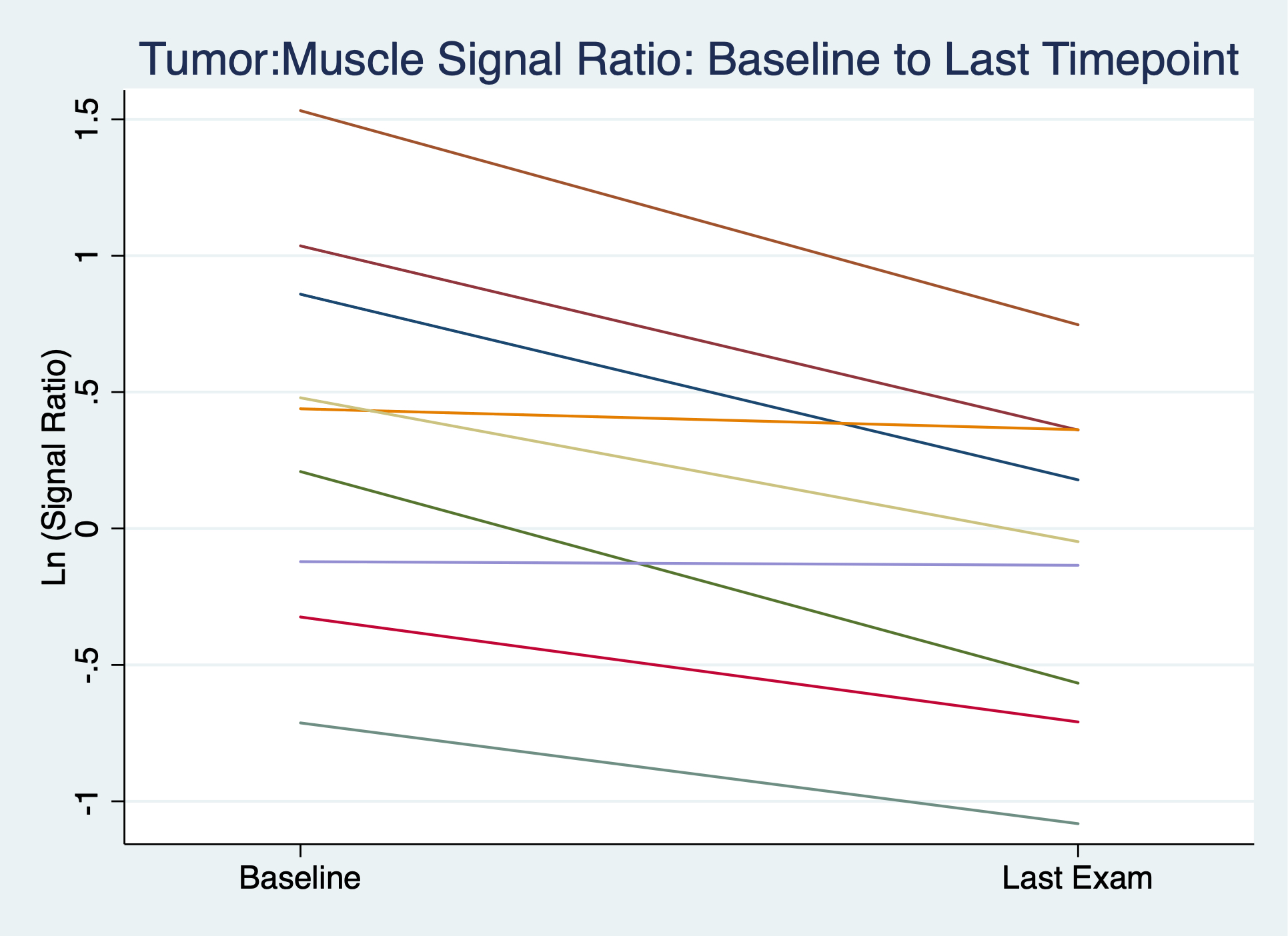

Supplement: Supplementary Figure 2 — Subject level (n=9) changes in tumor:muscle signal ratio from baseline to last exam, showing decrease for most subjects (mean -36%). [file Image_2.jpeg]

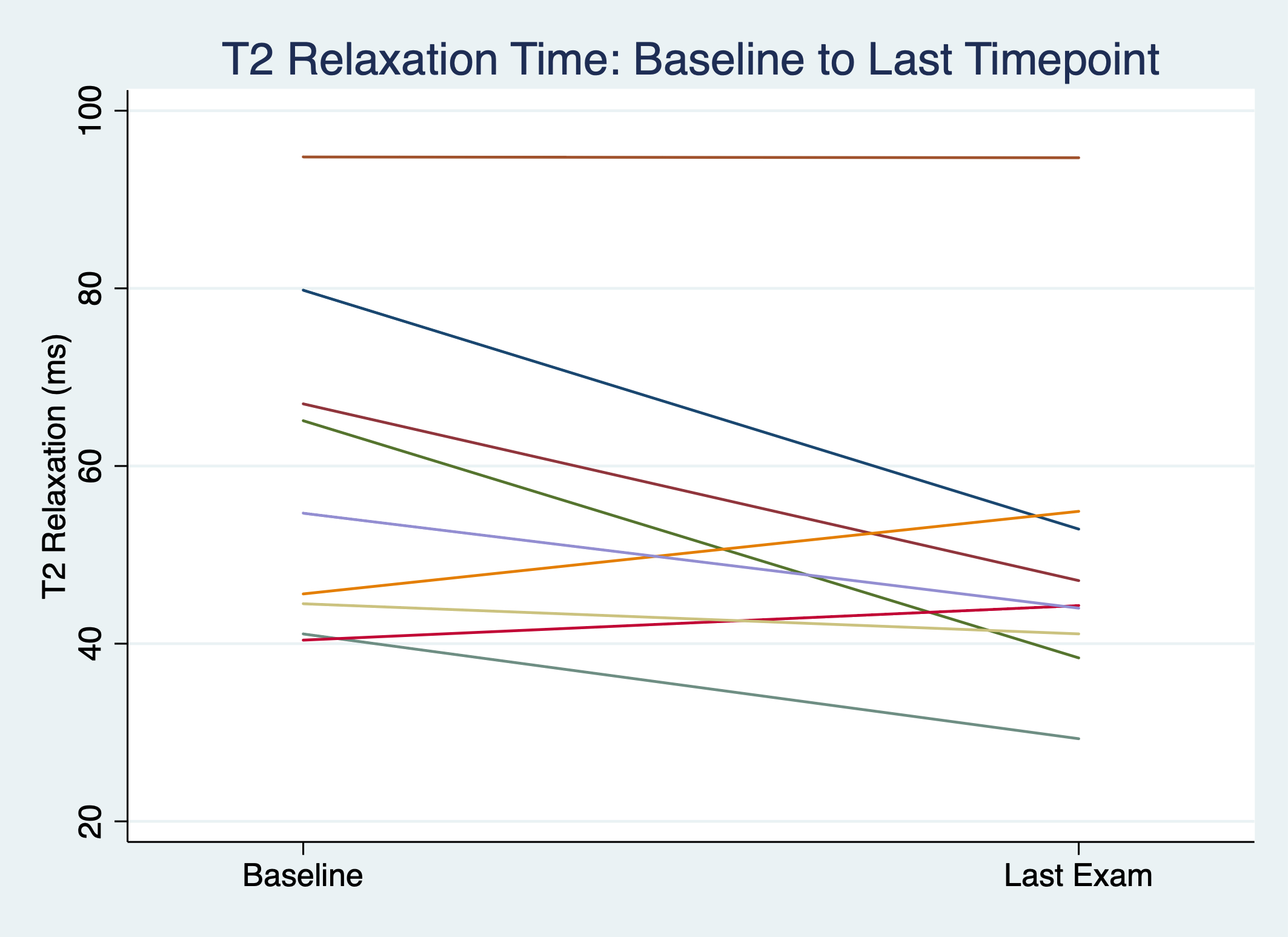

Supplement: Supplementary Figure 3 — Subject level (n=9) changes in T2 relaxation time from baseline to last exam, showing decrease for most subjects (mean -15%). [file Image_3.jpeg]

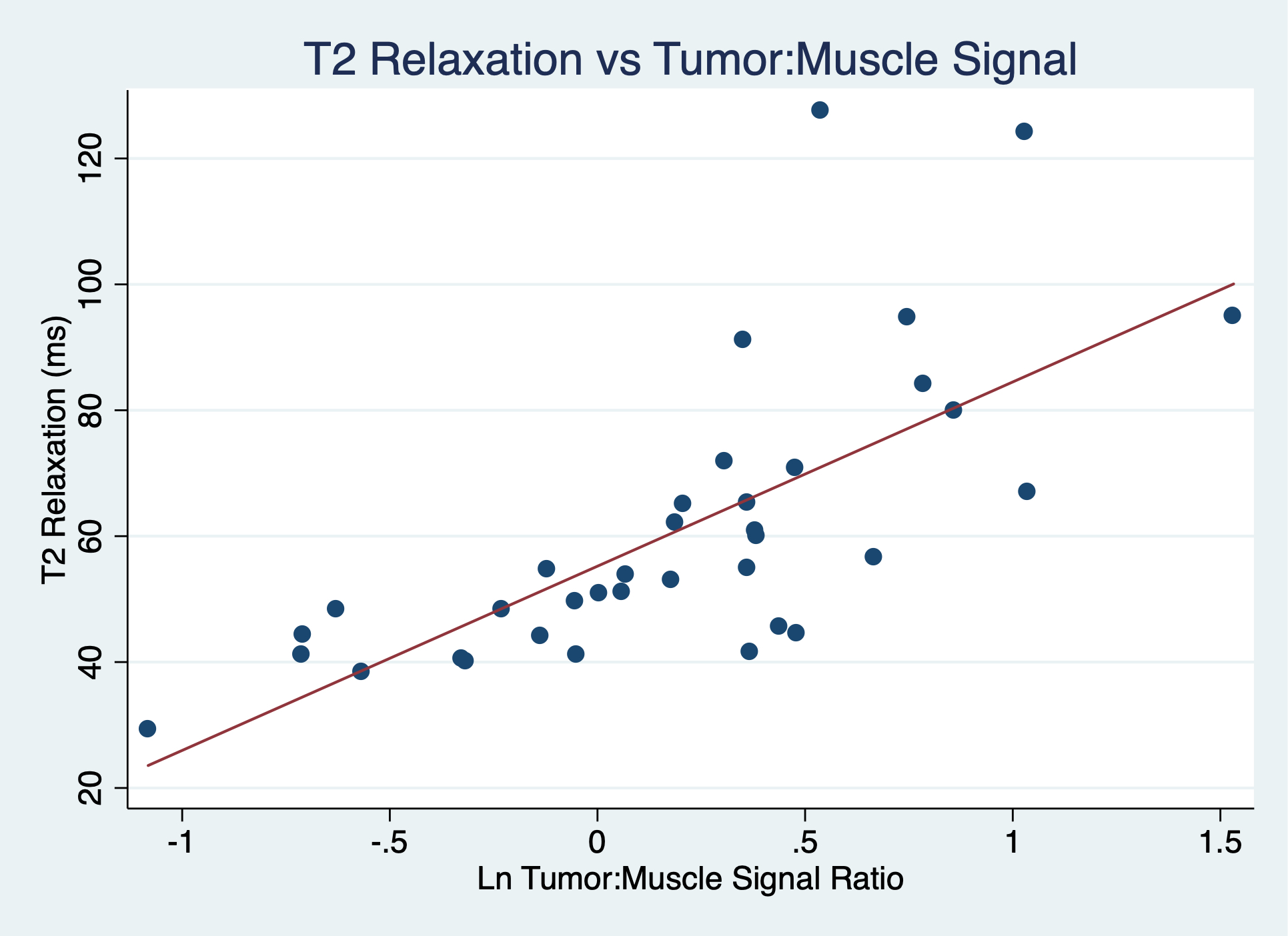

Supplement: Supplementary Figure 4 — Scatter plot depicting T2 Relaxation time vs tumor:muscle signal ratio across 36 time- point assessments shows good correlation (Pearson r =0.71, p < 0.001). [file Image_4.jpeg]
