# Supplementary material for: MRI T2 mapping assessment of T2 relaxation time in desmoid tumors as a quantitative imaging biomarker of tumor response: preliminary results
Source: Front Oncol. 2023 Dec 22;13:1286807. doi: 10.3389/fonc.2023.1286807 (PMC10766853; doi:10.3389/fonc.2023.1286807)
Supplement: Supplementary file 5 [file Table_1.pdf]

Supplemental Table: Location and signal intensities of muscle and tumor

ID	Observation	Location	Muscle	Tumor	Signal Ratio
1	1	abdominal wall	29.9	70.6	2.36
1	2	abdominal wall	40.4	58.4	1.45
1	3	abdominal wall	37.9	45.3	1.20
2	4	neck	83.2	234.5	2.82
2	5	neck	215.4	603.9	2.80
2	6	neck	235.9	336	1.42
2	7	neck	325.2	466.6	1.43
4	8	abdominal wall	149.7	203.5	1.36
5	9	chest wall	256.4	315.9	1.23
5	10	chest wall	176.2	258.9	1.47
5	11	chest wall	288.2	209.8	0.73
5	12	chest wall	287.5	163.1	0.57
6	13	thigh	46.8	72.6	1.55
6	14	thigh	180.9	218.2	1.21
6	15	thigh	113.6	221.3	1.95
6	16	thigh	177.1	259.6	1.47
6	17	thigh	170	244.2	1.44
7	18	calf	320.6	157.2	0.49
7	19	calf	244	82.7	0.34
8	20	chest wall	284.2	205.5	0.72
8	21	chest wall	252.7	201.1	0.80
8	22	chest wall	95.9	51.2	0.53
8	23	chest wall	107.1	52.7	0.49
9	24	abdominal wall	79.6	70.5	0.89
9	25	abdominal wall	131.4	124.8	0.95
9	26	abdominal wall	76.6	77	1.01
9	27	abdominal wall	179.1	190.3	1.06
9	28	abdominal wall	226.3	242.8	1.07
9	29	abdominal wall	147.5	128.9	0.87
10	30	chest wall	65.7	106.1	1.61
10	31	chest wall	82.8	78.9	0.95
11	32	neck	212.1	341.4	1.61
12	33	shoulder	15	69.4	4.63
12	34	shoulder	192.8	330.4	1.71
12	35	shoulder	40.6	89.2	2.20
12	36	shoulder	32.6	68.8	2.11

Supplemental table displays desmoid tumor location and measured raw signal intensities from fluid-sensitive sequences for calculating tumor:muscle signal ratios. One timepoint was missing fluid sensitive sequences, precluding calculation of a tumor:muscle signal ratio.
